# Can Higher Land Rentals Promote Soil Conservation of Large-Scale Farmers in China?

**DOI:** 10.3390/ijerph192315695

**Published:** 2022-11-25

**Authors:** Wang Ge, Shiyun Zhang, Yan Lu, Jiyu Jiang, Hui Jiang, Xiaona Cheng

**Affiliations:** 1 College of Economics and Management, Anhui Agricultural University, Hefei 230036, China; 2 Institute of Agricultural Economics and Development, Chinese Academy of Agricultural Sciences, Beijing 100081, China

**Keywords:** land rentals, soil conservation, land lease term, agricultural extension services, large-scale farmers

## Abstract

Based on theoretical analysis, this study empirically analyzes the mediating mechanism of how land rentals work on large-scale farmers to enhance soil conservation with survey data of 425 large-scale farmers in Shandong and Anhui Provinces, the main grain-producing regions of China, and further examines the moderating effect of agricultural extension services. The results show that: (1) The higher the land rentals, the greater the probability that large-scale farmers enhance soil conservation. (2) The mediating effect demonstrates that in a highly market-oriented rural land transfer market in China, the land lease term of large-scale farmers is longer with the increase of land rentals, thus motivating large-scale farmers to engage in soil conservation. (3) As shown by the moderating effect, agricultural extension services can further positively moderate the contribution of land lease term to large-scale farmers enhancing soil conservation. In order to encourage large-scale farmers to enhance soil conservation, on the one hand it is necessary to standardize the land transfer market and proactively guide large-scale farmers to extend the land lease term. On the other hand, it is indispensable to strengthen agricultural extension services and further broaden the access to soil conservation technologies for large-scale farmers.

## 1. Introduction

Soil, as the basis of agricultural production, serves as an important strategic resource to ensure national food security. Benefiting from the investment of modern production factors, such as chemical fertilizers and pesticides, China has created the miracle of feeding 22% of the world’s population with only 7% of the world’s farmland resources [1], making an important contribution to coping with the global food crisis. However, inevitably, China’s food production has also become heavily dependent on chemical fertilizers and pesticides. Currently, China has become the world’s largest consumer of chemical fertilizers and pesticides [2,3], which has brought about a series of environmental problems, such as soil compaction and acidification, reduced microbial activity, and heavy metal pollution, resulting a significant drop of soil quality [4]. From 2009 to 2015, the proportion of high-quality farmland in China decreased from 30% to 27%, while the proportion of medium-quality and low-quality farmland increased from 68% to 71% [5]. Therefore, how to guide farmers to adopt green agricultural technologies, reduce their dependence on chemical fertilizers and chemical pesticides, and improve soil quality, has become a very urgent practical problem.

In order to improve soil quality and reduce the consumption of chemical fertilizers and pesticides, the Ministry of Agriculture and Rural Affairs of China began to implement the zero increase action in the use of chemical fertilizers, and the zero increase action in the use of pesticides, in 2015. The former ensured a zero increase in the use of chemical fertilizers by promoting organic fertilizers, soil testing and formulated fertilization, and other technologies; the latter ensured zero increase in the use of pesticides by promoting biological pesticides, low-toxic pesticides, and others. Because organic fertilizers can increase soil organic matter and improve the activity of soil enzymes and microorganisms [6], soil testing and formula fertilization play an important role in effectively balancing soil nutrients and improving soil fertility [7]. Biological pesticides are far less toxic than traditional chemical pesticides and have no residue in the soil, which is a green technology beneficial to soil [8]. The Chinese government hopes to protect soil by promoting the above technologies to reduce the loss of chemical fertilizers and reduce pesticide residues.

However, it has been shown that farmers in developing countries are seemingly not concerned about soil conservation [9], and soil conservation technology is not widely used [10,11]. This is mainly due to the fact that soil conservation technologies requires more labor and money [12,13], and farmers in developing countries prefer to use chemical fertilizers and chemical pesticides to obtain short-term benefits [14]. As in many developing countries, farmers in China have low income levels and lack the impetus to carry out soil conservation [9]. At the same time, the Household Contract Responsibility System from the 1980s has resulted in the small farmland size of Chinese farmers, with serious farmland fragmentation [15], which is not conducive to the promotion of soil conservation technologies [16]. According to the Third National Land Survey, China’s per capita area of farmland is 0.09 hectares, which is only 34.62% of the world average [17].

Because the small-scale family operation model is no longer suitable for the modernization development of agriculture in China [16], since 2012 the Chinese government has taken promoting land transfer and developing moderate scale management as an important work [18]. It successively issued policies to strongly support large-scale farmers (according to the standard set by the Ministry of Agriculture and Rural Affairs of China, farmers who operate on at least 3.33 ha of farmland in the region of two-harvest-a-year are classed as large-scale farmers). By 2020, large-scale farmers with a land area of more than 3.33 hectare in China have reached 4.517 million. Compared with small-scale farmers, large-scale farmers operate a larger land, with a lower cost of applying soil conservation technologies [16], a lower degree of time preference, and are more sensitive to future benefits [19]. Thus, theoretically, large-scale farmers are more likely to apply soil conservation technologies in large numbers when land management is stable.

**Figure 1:**
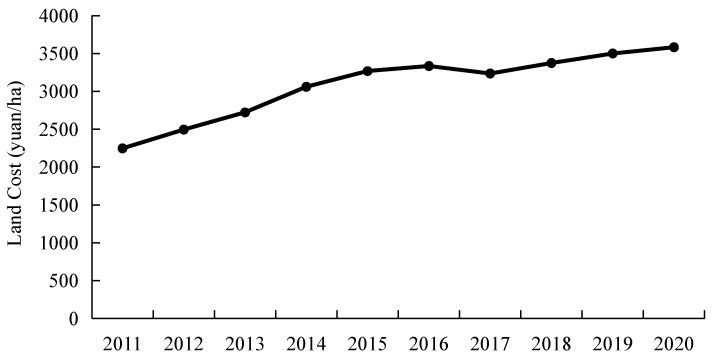
Average land cost of wheat, rice, and maize in China from 2011–2020.

Meanwhile, under policy incentives, more and more farmers and agricultural entrepreneurs began to transfer-in a large percentage of land, resulting in a significant increase in land rentals, and an increasing degree of marketization of land rentals in China [20]. According to China’s largest land trading platform (www.tuliu.com), from 2014 to 2017 the rent of irrigated land increased 163 yuan/ha per year on average, and the rent of rain-fed land increased 611 yuan/ha per year on average [21]. Rental has become the biggest cost of agricultural production. According to macroscopic statistics, the average land cost of the most three important grains (wheat, rice and maize) in China increased from 2246.25 yuan/ha to 3582.3 yuan/ha from 2011 to 2020, an increase of 59.48% (Figure 1). In the face of rising land rentals, are large-scale farmers motivated to adopt soil conservation technologies?

## 2. Review of the Research

Soil conservation belongs to both technology adoption and long-term investment. First, from the perspective of technology itself, soil conservation technologies have the characteristics of long investment cycle and complicated operation, and its adaptability requirements for agricultural machinery are relatively high [22]. Farmers in most developing countries have low incomes, and it is not economical for small-scale farmers to purchase agricultural machinery [23], which may limit the promotion of soil conservation technologies. However, since entering the 21st century, the level of agricultural mechanization in China has increased significantly, one is that agricultural machinery subsidies motivate farmers to purchase agricultural machinery [23], and the other is that the development of outsourced mechanical services has cleared obstacles for farmers to adopt soil conservation technologies [24]. From 2000 to 2018, the total power of agricultural machinery in China increased from 525.74 million kilowatts to 1003.77 million kilowatts [24]. Moreover, according to the data of the Ministry of Agriculture and Rural Affairs of China, in 2022 the mechanization rates of the wheat, rice, and maize in China has exceeded 97%, 90%, and 85%, respectively.

Second, from a cost-benefit perspective, rising land rentals can have an important impact on farmers’ adoption of soil conservation technologies. However, there is considerable controversy over the impact of land rentals on large-scale farmers to enhance soil conservation. Many studies show that land rentals significantly inhibit farmers to enhance soil conservation [25,26,27,28]. Recent studies on China, however, concludes that higher land rentals nevertheless favor soil conservation. Based on a study of farm household survey data in Guangxi Province, China, Qian et al. [29] reveals a phenomenon that the higher the land rental is, the more likely farmers are to adopt soil formula fertilization and straw return technologies to improve soil quality. Based on data from the China Household Finance Survey (CHFS), Li et al. [30] found that farmers who pay land rentals are more likely to undertake soil conservation investments than those who pay zero rental. Li et al. [31] found that land rental reflects soil quality, and the empirical results show that for every 1% increase in land rentals, farmers’ investment of organic fertilizer increases by 1.7%.

Third, from the perspective of property rights, stable land tenure is an important factor to promote farmers’ long-term investment [32,33,34], and unstable land tenure is not conducive to farmers’ adoption of soil conservation technologies. Land has the property of asset specificity and soil conservation is a long-term investment, which can have a “hold-up” effect when large-scale farmers invest in soil conservation and leads to the opportunism of land transfer-out farmers. Therefore, it is very important for the government to develop a sound regulatory system. In the early land transfer market in China, the system is not perfect, of which oral contracts and informal transfers of land transactions are widespread, combined with unstable land management, as well as frequently occurred land transfer contract disputes [35]. However, an important change is that the land transfer market is becoming more and more standardized in China. The government has adopted measures such as establishing a trading platform for rural collective assets, and implementing a performance guarantee insurance for the transfer of rural land management rights. This has driven the continuous improvement of related systems, while promoting the market-based trading of land management rights. In the formal land transfer market, the pressure of rising land rentals year by year makes large-scale farmers more willing to sign long-term contracts [27], and this adjustment of contract land lease term is conducive to promoting large-scale farmers’ soil conservation. However, the existing literature all separate land rentals from the land lease term, ignoring the possible causal relationship between the two.

Fourth, there is a problem that existing studies on the influence of land rentals on farmers’ soil conservation have neglected the moderating effect of agricultural extension services. Agricultural extension services can give an impulse to farmers’ adoption of soil conservation technologies. Currently, Chinese farmers’ soil conservation methods mainly include adopting organic fertilizers instead of chemical fertilizers, using soil testing and formulated fertilization, and applying biological pesticides instead of chemical pesticides, etc. Therefore, for farmers with limited access to information, these new green agricultural technologies rely heavily on agricultural extension services. Qiao et al. [36] pointed out that agricultural extension services can effectively improve farmers’ ecological cognition and promote farmers’ organic fertilizer investments. Therefore, in studying the influence of land rentals on farmers’ soil conservation, it is logical to consider the moderating effect of agricultural extension services and to study land rentals, land lease term, agricultural extension services and large-scale farmers’ soil conservation in a unified theoretical framework.

In this study, based on the theoretical analysis, the survey data of 425 large-scale farmers in Shandong and Anhui provinces, the main grain-producing regions in China, are used to empirically study the mechanism of the effect of land rentals on large-scale farmers’ soil conservation. At the same time, we further study the moderating effect of agricultural extension services, and finally propose policy recommendations to enhance the soil conservation of large-scale farmers. The innovations of this study mainly lie in the following points. First, as the first study on the effect of land rentals on the land lease term of large-scale farmers’ land management right, this study shows that higher land rentals promote long-term leasing on large-scale farmers in the Chinese market-oriented rural land transfer market. Second, for the first time, the mediating effect of the effect of land rentals on large-scale farmers’ soil conservation is studied from the perspective of land lease term. The study shows that the land lease term plays a mediating effect in the effect of land rentals on large-scale farmers’ soil conservation. It turns out to be that the higher the land rental, the longer the land lease term of the large-scale farmer, and the higher the probability of soil conservation. Third, this study is the first to include land rentals, land lease term, agricultural extension services, and large-scale farmers’ soil conservation into a unified theoretical framework for research, through which the moderating effect played by agricultural extension services is further investigated. According to the research results, agricultural extension services are conducive to further positively moderating the land lease term on large-scale farmers’ soil conservation.

## 3. Theoretical Analysis

### 3.1. Analysis of the Direct Effect of Land Rentals on Large-Scale Farmers’ Soil Conservation

Large-scale farmers, as the subjects specializing in grain production, pursue profit maximization because it is their primary decision-making goal. However, in recent years, the cost of grain production in China has continued to rise while the “ceiling” effect of import prices bolster [37], squeezing the profit margin of grain production. According to statistics, from 2011 to 2020 the average labor cost of wheat, rice, and maize in China increased from 4245.75 yuan/ha to 6191.4 yuan/ha, an increase of 45.83%; the material cost increased from 5375.4 yuan/ha to 7020.15 yuan/ha, an increase of 30.60%. However, from the point of view of unit area revenue, the average output value of wheat, rice, and maize, three major food crops, increased from 15,223.8 yuan/ha to 17,500.95 yuan/ha from 2011 to 2020, an increase of only 14.88%. In particular, the profit of grain production will further decline under the continuous rise of land rentals.

The improvement of the living standard of the population makes consumers pay more attention to the quality of agricultural products, so the demand for high-quality, safe agricultural products is increasing. Compared with food produced with chemical fertilizers and pesticides, green and pollution-free food produced with organic fertilizers and biological pesticides is sold at a higher market price. In China, organic agricultural products are more expensive than the price of regular agricultural products by over 30% [38]. Therefore, in order to obtain higher returns, large-scale farmers are more willing to use organic fertilizers instead of chemical fertilizers and biological pesticides instead of chemical pesticides, which not only can guarantee the returns of large-scale farmers, but also can further improve soil quality. Thus, in a highly market-oriented rural land transfer market in China, the higher the land rentals, the more willing large-scale farmers are to adopt soil conservation technologies. Accordingly, this study proposes research hypothesis 1.

**Hypothesis** **1** **(H1).** *Higher land rentals can promote the adoption of soil conservation technologies by large-scale farmers*.

### 3.2. Analysis of the Indirect Effect of Land Rentals on Large-Scale Farmers’ Soil Conservation through Land Lease Term

First, higher land rentals indicate better soil quality and more long-term gains [31]. Land rentals reflect soil quality to some extent. Large-scale farmers are rational economic people, and one of their goals is to maximize profit. Because of the existence of the magnitude effect [39], farmers who operate larger land areas have lower time preferences and are more sensitive to future gains [19]. Soil conservation is a long-term investment with high investment costs, long recovery cycles, and high future benefits. Therefore, the higher the land rentals, the more willing large-scale farmers are to sign long-term contracts.

Long-term and stable land management rights incentives are conducive to the investment of large-scale farmers in soil conservation, while existing studies generally agree that property rights incentives are an important factor in promoting farmers’ investment [6,32,33]. With the continuous improvement of China’s land transfer market, land lease contracts are increasingly standardized. Long-term land lease contracts ensure the stability of large-scale farmers’ land management right and prevent opportunism, which in turn drives large-scale farmers to employ soil conservation. Therefore, the rise in land rentals enhances large-scale farmers’ soil conservation investments through the long-term land lease term. Accordingly, this study proposes research hypothesis 2.

**Hypothesis** **2** **(H2).** *The land lease term of large-scale farmers is longer with the increase of land rentals, thus motivating large-scale farmers to engage in soil conservation*.

### 3.3. Analysis of the Moderating Effect of Agricultural Extension Services on Large-Scale Farmers’ Soil Conservation Investments Affected by Land Lease Term

**Figure 2:**
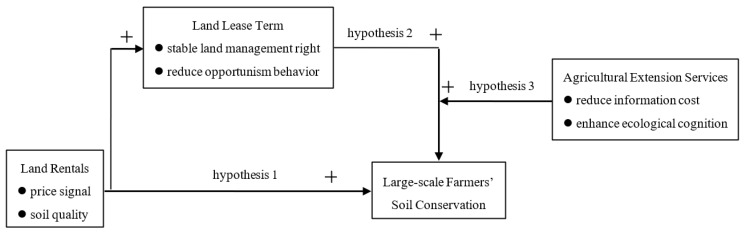
Conceptual diagram.

Agricultural extension services can further enhance soil conservation for large-scale farmers. Agricultural extension services are one of the main ways for Chinese farmers to acquire new technologies and knowledge. Wossen et al. [40] pointed out that agricultural extension services reduce farmers’ information seeking costs, provide channel support for their adoption of green production technologies, and increase farmers’ willingness to adopt new technologies. Thus, agricultural extension services play a positive role in increasing the likelihood of large-scale farmers to adopt green production technologies such as organic fertilizers, soil testing and formulated fertilization, and biological pesticides. On the other hand, agricultural extension services can improve the cognition level of large-scale farmers. Qiao et al. [36] pointed out that access to agricultural extension services can improve the ecological cognition level of farmers, i.e., it strengthens farmers’ awareness of environmental pollution caused by excessive fertilizer application, which in turn promotes farmers to adopt organic fertilizers instead of chemical fertilizers to improve the environment. In other words, agricultural extension services can enhance soil conservation by raising the ecological cognition level of large-scale farmers. Therefore, while land rentals enhance large-scale farmers’ soil conservation investments through the land lease term, agricultural extension services can further play a positive moderating effect. Accordingly, this study proposes research hypothesis 3, Figure 2.

**Hypothesis** **3** **(H3).** *Agricultural extension services can play a positive facilitating effect in moderating the land lease term on the large-scale farmers’ soil conservation*.

## 4. Data and Methods

### 4.1. Data Source

The data in this paper are obtained from the research team’s special research on large-scale farmers in Shandong and Anhui provinces of China. The research in Shandong Province was conducted in 2019, and the research area included eight large grain-producing counties in three prefecture-level cities, including Dezhou City, Liaocheng City, and Heze City. The research in Anhui Province was conducted in 2017, and the research area was 11 large grain-producing counties in five cities, including Bengbu City, Suuzhou City, Chuzhou City, Fuyang City, and Bozhou City. The research team selected two random townships in each county on the basis of sampling the sample counties, and each township took about 12 large-scale farmers. The research method adopted was through one-on-one interviews between the researcher and large-scale farmers, with a total of 448 large-scale farmers investigated. Some samples were excluded due to the lack of relevant indicators, land areas less than 3.33 ha and other reasons. Finally, retained a valid sample of 425 large-scale farmers. Among them, 237 large-scale farmers were retained in Shandong Province and 188 large-scale farmers in Anhui Province, with a sample efficiency of 94.87%.

### 4.2. Variable Setting

#### 4.2.1. Dependent Variable

The dependent variable of this paper is whether to adopt soil conservation technologies, and the widely used soil conservation technologies include organic fertilizer, soil testing and formulated fertilization, and biological pesticides. Therefore, when large-scale farmers adopt any of the above three kinds, we consider that large-scale farmers adopt soil conservation technologies.

#### 4.2.2. Core Independent Variable

The core independent variable of this paper is land rentals, which specifically refers to the amount of money paid by the large-scale farmers to a unit area of land.

#### 4.2.3. Mediating Variable

The mediating variable in this paper is the land lease term, which specifically refers to the number of years in the land lease contract signed by the large-scale farmers.

#### 4.2.4. Moderating Variable

The moderating variable in this paper is agricultural extension services, specifically the number of agricultural technology training attended by the large-scale farmers in that year. The reason why this is the moderating variable, is because its participation in agricultural technology training is one of the main ways for large-scale farmers to obtain soil conservation technologies.

#### 4.2.5. Control Variables

In terms of the control variables, in addition to considers individual characteristics, family characteristics and operation characteristics in controlling the heterogeneity of large-scale farmers; for example, the age and education of head of household, the number of agricultural laborers, average output value per ha, average input per ha, land area, agricultural subsidies. We also consider whether the head of household is village cadre and joins a cooperative or not. Because village cadres are a social status [41], it is more likely for them to adopt soil-conservation technologies. Joining a cooperative makes them obtain more technologies [16]. In addition, terrain and regional variable are considered. Plain areas are conducive to the application of large-scale agricultural machinery and promote soil-conservation technologies promotion. Regional variables reflect the differences in economic and social development level between regions, and economic development in Shandong Province is better than that in Anhui Province. In order to weaken the influence of outliers on the regression results, this study conducted 1% and 99% percentile tail reduction processing on the relevant continuous variables. The variable descriptions and descriptive statistics are shown in Table 1.

### 4.3. Model Selection

#### 4.3.1. Probit Model

Given that the dependent variable in this paper is a binary dummy variable, the baseline regression model adopts the probit model.

(1) $$Y=cX+dR+e_{1}$$

The model is set as follows: where *Y* represents the dependent variable that whether to adopt soil conservation technologies; *X* represents the core independent variable the land rental; *R* represents the control variables; c is the total effect of *X* on *Y*, *d* is the coefficient of control variables; and *e*_1_ is the residual term.

#### 4.3.2. Mediating Effect Model

Mediating effect model is commonly used to reveal internal mechanisms in causality that can clarify complex causality and is widely adopted in empirical studies [42,43]. Therefore, this study continues to build a mediating effect model based on Equation (1), with the aim of analyzing the mechanism of land rentals on large-scale farmers’ soil conservation.

(2) $$M=aX+dR+e_{2}$$

(3) $$Y=c’X+bM+dR+e_{3}$$

where *M* is the mediating variable land lease term; *c’* is the direct impact of *X* on *Y* after controlling the effect of the mediating variable *M*. The product of *a* and *b*, *ab*, denotes the mediating effect after the mediating variable *M*. *e*_2_ and *e*_3_ are the residual terms. In the case that the effects of *X* in (1) and (2) are significant and the effects of *X* and *M* in (3) are significant, but the absolute value of *c’* is smaller than the absolute value of *c*, it indicates a partial mediating effect; In the case that the effects of *M* in (3) are significant but the effects of *X* are not significant, it indicates a full mediating effect.

#### 4.3.3. Moderating Effect Model

The moderating effect model is widely used, and the interaction between two variables can be analyzed by introducing interaction terms [44,45]. Therefore, the following moderating effect model is established to analyze the moderating effects of agricultural-extension-services:

(4) $$Y=c_{1}X+b_{1}M+a_{1}U+dR+e_{4}$$

(5) $$Y=c_{2}X+b_{2}M+a_{2}U+a_{3}MU+dR+e_{5}$$

where *U* represents the moderating variable agricultural extension services, *MU* represents the interaction term between land lease term and agricultural extension services, and *e*_4_ and *e*_5_ represent the residual term. If the coefficients *a*_1_ and *a*_3_ are significant, it means that agricultural extension services have a significant moderating effect.

## 5. Results

### 5.1. Direct Effect of Land Rentals on Large-Scale Farmers’ Soil Conservation

Before regression, this paper uses variance inflation factor (VIF) to test the variables for multicollinearity. According to the test results, the maximum variance inflation factor of each variable is 1.83, and the mean value is 1.24, indicating that there is no multicollinearity problem. With the help of the Stata15 software, the probit model was developed. On this basis, the direct effect of land rentals on large-scale farmers’ soil conservation was estimated in this paper, and the regression results are shown in Table 2.

Regression (1) shows the results of separate regressions of the effect of land rentals on large-scale farmers’ soil conservation. The coefficient of “Land Rentals” is 0.637, which is significant under the 1% statistical level, indicating that the higher the land rentals, the higher the probability that large-scale farmers engage in soil conservation. Given that this result is consistent with expectations, it has value for further analysis.

Regression (2) shows the results of the model regression with the inclusion of the control variables. The results show that the coefficient of “Land Rentals” is 0.537, which is significant under the 5% statistical level, indicating that the higher the land rentals, the higher the probability of soil conservation by large-scale farmers, after controlling other variables. Given that this result is consistent with the expectation of research hypothesis 1, which states that higher land rentals enhance soil conservation by large-scale farmers, research hypothesis 1 is validate.

Among the control variables, the coefficient of “Average Output Value Per Ha” is 0.227. The result shows that it is significant under the 5% statistical level, indicating that the higher the land output rate, the higher the probability that large-scale farmers will engage in soil conservation. Given that the coefficient of “Agricultural Subsidies” is 2.236 and is significant under 5% statistical level, agricultural subsidy income helps to enhance soil conservation among large-scale farmers. The coefficient of “Education Level” is 0.232 and significant under 5% statistical level, indicating that the higher the education level of the large-scale farmers, the higher the probability of adopt soil conservation technologies. Since the coefficients of variables such as “Average Input Per Ha” “Number of Agricultural Labor”, “Age”, “Land Area”, “Village Cadre or not”, “Join a Cooperative or not”, and “Terrain”, do not pass the statistical significance test, it indicates that the above-mentioned individual characteristics, family characteristics, and operating characteristics, do not have significant effects on how large-scale farmers enhance soil conservation.

### 5.2. Mediating Effect Test of Land Lease Term

The stepwise regression method for mediating effect test of land lease term was adopted with the aim of investigating the mechanism underlying the effect of land rentals on large-scale farmers’ soil conservation. The regression results are shown in Table 3. Among them, regression (3) is the regression result of the effect of land rentals on the land lease term of large-scale farmers, and regression (4) is the regression result of the effect of land rentals and land lease term on large-scale farmers’ soil conservation.

According to regression (2) and regression (3), the coefficients of “Land Rentals” are 0.537 and 2.314, respectively, both of which are significant under the 5% statistical level, indicating that land rentals have a significant effect on soil conservation and the land lease term for large-scale farmers. In regression (4), the coefficients of “Land Rentals” are 0.450 and the coefficient of “Land Lease Term” is 0.040, both of which are significant under the 5% statistical level, and the coefficient of “Land Rentals” is smaller than the coefficient in regression (2). According to these results, in the process of land rentals influencing soil conservation of large-scale farmers, land lease term has a partially mediating effect. In other words, land rentals not only directly enhance soil conservation by large-scale farmers, but also increase the land lease term, which indirectly enhances large-scale farmers to intensify soil conservation. In this way, the research hypothesis 2 is verified.

### 5.3. Robustness Test

To ensure the robustness of the regression results, this paper further tests the mediating effects by using Sobel test and Bootstrap test, and the results are shown in Table 4.

In the Sobel test, the coefficients of “Land Rentals” in Step 1 and Step 2 are 0.155 and 2.314, respectively, and are significant at least under the 5% statistical level. The coefficients of “Land Rentals” in Step 3 are 0.011 and the coefficient of “Land Lease Term” is 0.129, both of which pass the significance test. The z value is 1.984, which is greater than the critical value of 1.96, and the mediating effect accounts for 17.05%. In the Bootstrap test, given that the lower limit of the 95% confidence interval is 0.007 and the upper limit is 0.065, excluding 0, the mediating effect works. The fact that the above two tests of mediating effects both indicate that the land lease term plays a mediating effect in the influence of land rentals on soil conservation by large-scale farmers, and the higher the land rentals, the longer the land lease term of large-scale farmers and the higher the probability of soil conservation.

### 5.4. Moderating Effect Test of Agricultural Extension Services

Further, a moderating effect model was developed to examine the moderating effect of agricultural extension services in the effect of land lease term on soil conservation of large-scale farmers. The regression results are shown in Table 5.

Regression (5) is the result of introducing agricultural extension services. The coefficient of “Agricultural Extension Services” is 0.087, which is significant under the 10% statistical level, indicating that agricultural extension services can positively enhance large-scale farmers to carry out soil conservation.

Regression (6) shows the regression results of introducing the interaction terms of “Agricultural Extension Services” and “Land Lease Term”. Considering that the direct introduction of the interaction term may cause the problem of multicollinearity, this paper takes a centralized treatment of the variables. From the results, it can be seen that the coefficient of the interaction term between “Agricultural Extension Services” and “Land Lease Term” is 0.022, which is significant under the 5% statistical level. This indicates that while land rentals enhance soil conservation of large-scale farmers through land lease term, agricultural extension services can positively moderate the promotion effect of land lease term on soil conservation of large-scale farmers. Therefore, the research hypothesis 3 is verified.

## 6. Conclusions and Implications

Since China is a country with a population of 1.4 billion, agriculture plays a dominant role and the strategic significance of farmland resources is extremely important. The Chinese government proposes to take “tougher” measures (measures are effective and useful, with accountability and clear rewards and punishments) to implement the strictest soil conservation system. Given that farmers are the micro subjects of soil conservation and the direct beneficiaries of soil quality improvement, how to boost the subjective initiative of farmers to enhance soil conservation is of great significance.

Based on theoretical analysis, this study empirically analyzes the mediating mechanism of how land rentals work on large-scale farmers to enhance soil conservation, with survey data of 425 large-scale farmers in Shandong and Anhui provinces, the main grain-producing regions of China, and further examines the moderating effect of agricultural extension services. We find that: (1) higher land rentals are beneficial in promoting the adoption of soil conservation technologies by large-scale farmers. This is consistent with the conclusion by Li et al. [31]. On the one hand, land rentals act as a price signal, and higher land rentals suggests that land transfer-in farmers are generally optimistic about income expectations and facilitate the adoption of soil conservation technologies by large-scale farmers. On the other hand, higher land rentals indicate better land quality [31], and are more conducive to promoting soil conservation by large-scale farmers. (2) The mediating effect suggests that land lease term plays a partial mediating role in land rentals affecting soil conservation by large-scale farmers. In other words, in the current land transfer market in rural China, with a higher degree of marketization, the higher the land rentals, the longer the land lease term of large-scale farmers, which then motivates large-scale farmers to carry out soil conservation. This is a useful addition to the existing literature. (3) The agricultural extension services favor the promotion of soil conservation by large-scale farmers, which is consistent with Qiao et al. [36]. (4) The moderating effect suggests that land rentals promote soil conservation input by large-scale farmers through land lease term, while agricultural extension services can positively adjust the promoting effect of land lease term on large-scale farmers’ soil conservation. (5) The higher the agricultural subsidies, the more enthusiastic large-scale farmers are to adopt soil conservation technologies. (6) The higher the education level of large-scale farmers, the higher the probability of adopting soil conservation technologies.

The above findings have important policy implications for the majority of developing countries: (1) It is necessary to further improve the land transfer market, because sound institutional development can prevent land transfer disputes, avoid opportunism in land transfer, and thus encourage large-scale farmers to enhance soil conservation. (2) It is necessary to actively guide large-scale farmers to practice long-term land transfer and extend the land lease term, which is of positive significance to play the incentive effect of stable property rights and encourage large-scale farmers to increase their investment in soil conservation. (3) It is necessary to strengthen agricultural extension services. On the one hand, the Ministry of Agriculture and Rural Affairs can increase the strength of agricultural extension services and carry out regular and systematic agricultural technologies training to provide technical support for large-scale farmers’ soil conservation investments. On the other hand, it is suggestive to use the power of agricultural enterprises, cooperatives, and other social organizations, to broaden the access to soil conservation, such as organic fertilizers, biological agriculture, and soil testing and formulated fertilization, for large-scale farmers.

## Figures and Tables

**Table 1:** Description of variables and descriptive statistics.

Items	Variables	Definition	Mean	S.D.	Min	Max
Dependent variable	Whether to Adopt Soil Conservation technologies	0 = No; 1 = Yes	0.739	0.440	0	1
Core independent variable	Land Rentals	Amount paid by large-scale farmers for transfer-in 1 ha of farmland (yuan)	1.049	0.346	0.15	2.10
Mediating variable	Land Lease Term	Number of years in the land transfer contract signed by the large-scale farmer (years)	7.776	4.978	1	30
Moderating variable	Agricultural Extension Services	Number of times large-scale farmers participated in agricultural technologies training in the year(times)	2.427	1.611	0	5
Control variables	Average Output Value Per Ha	Grain output value per hectare (yuan)	2.513	0.745	0.860	5.717
	Average Input Per Ha	Sum of material cost and labor cost for large-scale farmers to produce 1 hectare of grain (yuan)	0.777	0.204	0.105	2.40
	Agricultural Subsidies	Income from agricultural subsidies received by large-scale farmers for 1 hectare (yuan)	0.060	0.099	0	0.495
	Number of Agricultural Labor	Total number of family agricultural labor of large-scale farmers	2.271	0.969	1	7
	Age	Actual age of large-scale farmers (years)	47.25	7.342	29	70
	Education Level	0 = No education; 1 = Elementary School Education; 2 = Junior High School Education; 3 = High School Education; 4 = High School Education or Above	2.351	0.822	0	4
	Land Area	Actual area of land operated by large-scale farmers (hectares)	23.594	36.346	3.333	366.667
	Village Cadre or not	0 = No; 1 = Yes	0.256	0.437	0	1
	Join a Cooperative or not	0 = No; 1 = Yes	0.501	0.501	0	1
	Terrain	0 = Plain; 1 = Hilly	0.071	0.256	0	1
	Region	0 = Anhui; 1 = Shandong	0.558	0.497	0	1

**Table 2:** Regression results of land rentals on large-scale farmers’ soil conservation.

Variables	Regression (1)		Regression (2)	
	Coefficient	Robust Standard Error	Coefficient	Robust Standard Error
Land Rentals	0.637 ***	0.190	0.537 **	0.211
Average Output Value Per Ha			0.227 **	0.102
Average Input Per Ha			0.246	0.348
Agricultural Subsidies			2.236 **	1.013
Number of Agricultural Labor			−0.062	0.069
Age			0.004	0.010
Education Level			0.232 **	0.096
Land Area			−0.000	0.003
Village Cadre or not			0.032	0.165
Join a Cooperative or not			−0.030	0.151
Terrain			0.300	0.308
Region			0.019	0.182
Constant	−0.012	0.204	−1.379 *	0.709
Observations	425		425	
Log pseudo likelihood	−238.634		−225.943	
Wald chi^2^	11.25 ***		37.81 ***	
Prob > chi^2^	0.0008		0.0002	
Pseudo R^2^	0.0223		0.0743	

Note: *, ** and *** indicate passing the test at the significance levels of 10 %, 5%, and 1%, respectively.

**Table 3:** Regression results of mediating effect model.

Variables	Regression (3)		Regression (4)	
	Coefficient	Robust Standard Error	Coefficient	Robust Standard Error
Land Rentals	2.314 **	0.915	0.450 **	0.209
Land Lease Term			0.040 **	0.016
Average Output Value Per Ha	−0.705 **	0.340	0.261 **	0.102
Average Input Per Ha	−2.012 *	1.130	0.330	0.355
Agricultural Subsidies	0.992	2.749	2.093^**^	1.009
Number of Agricultural Labor	−0.184	0.211	−0.064	0.069
Age	0.062 *	0.037	0.001	0.010
Education Level	0.315	0.382	0.225 **	0.096
Land Area	0.022 ***	0.007	−0.001	0.003
Village Cadre or not	−1.613 ***	0.536	0.089	0.167
Join a Cooperative or not	0.760	0.492	−0.059	0.154
Terrain	3.920 ***	1.061	0.149	0.311
Region	2.913 ***	0.566	−0.091	0.191
Constant	3.077	2.290	−1.473 **	0.701
Observations	425		425	
Log pseudo likelihood			−222.630	
Wald chi^2^/F	5.75 ***		42.60 ***	
Prob > chi^2^	0.0000		0.0001	
Pseudo R^2^	0.1652		0.0878	

Note: *, ** and *** indicate passing the test at the significance levels of 10 %, 5%, and 1%, respectively.

**Table 4:** Results of Sobel test and Bootstrap test.

Variables	Step 1		Step 2		Step 3		Sobel Test	Percentage of Mediating Effect (%)	Bootstrap Test (Confidence Interval)
	Coef	Std Err	Coef	Std Err	Coef	Std Err			
Land Rentals	0.155 **	0.067	2.314 ***	0.716	0.011 **	0.005	1.984 **	17.05	[0.007, 0.065]
Land Lease Term					0.129 *	0.067			

Note: *, ** and *** indicate passing the test at the significance levels of 10 %, 5%, and 1%, respectively. Bias-corrected nonparametric percentile Bootstrap method was used and repeated sampling 5000 times.

**Table 5:** Regression results of moderating effect model.

Variables	Regression (5)		Regression (6)	
	Coefficient	Robust Standard Error	Coefficient	Robust Standard Error
Land Rentals	0.476 **	0.212	0.505 **	0.217
Land Lease Term	0.041 ***	0.016	0.051 ***	0.016
Agricultural Extension Services	0.087 *	0.046	0.104 **	0.048
Interaction term			0.022 **	0.010
Control Variables	Controlled		Controlled	
Observations	425		425	
Log pseudo likelihood	−220.684		−218.195	
Wald chi^2^	48.08 ***		51.38 ***	
Prob > chi^2^	0.0000		0.0000	
Pseudo R^2^	0.0958		0.1060	

Note: *, ** and *** indicate passing the test at the significance levels of 10 %, 5%, and 1%, respectively.

## Data Availability

Not applicable.
